# Supplementary material for: CD229 CAR T cells eliminate multiple myeloma and tumor propagating cells without fratricide
Source: Nat Commun. 2020 Feb 7;11:798. doi: 10.1038/s41467-020-14619-z (PMC7005855; doi:10.1038/s41467-020-14619-z)
Supplement: Supplementary file 1 — Supplementary Information [file 41467_2020_14619_MOESM1_ESM.pdf]

CD229 CAR T cells eliminate multiple myeloma and tumor propagating cells without fratricide

Radhakrishnan et al.

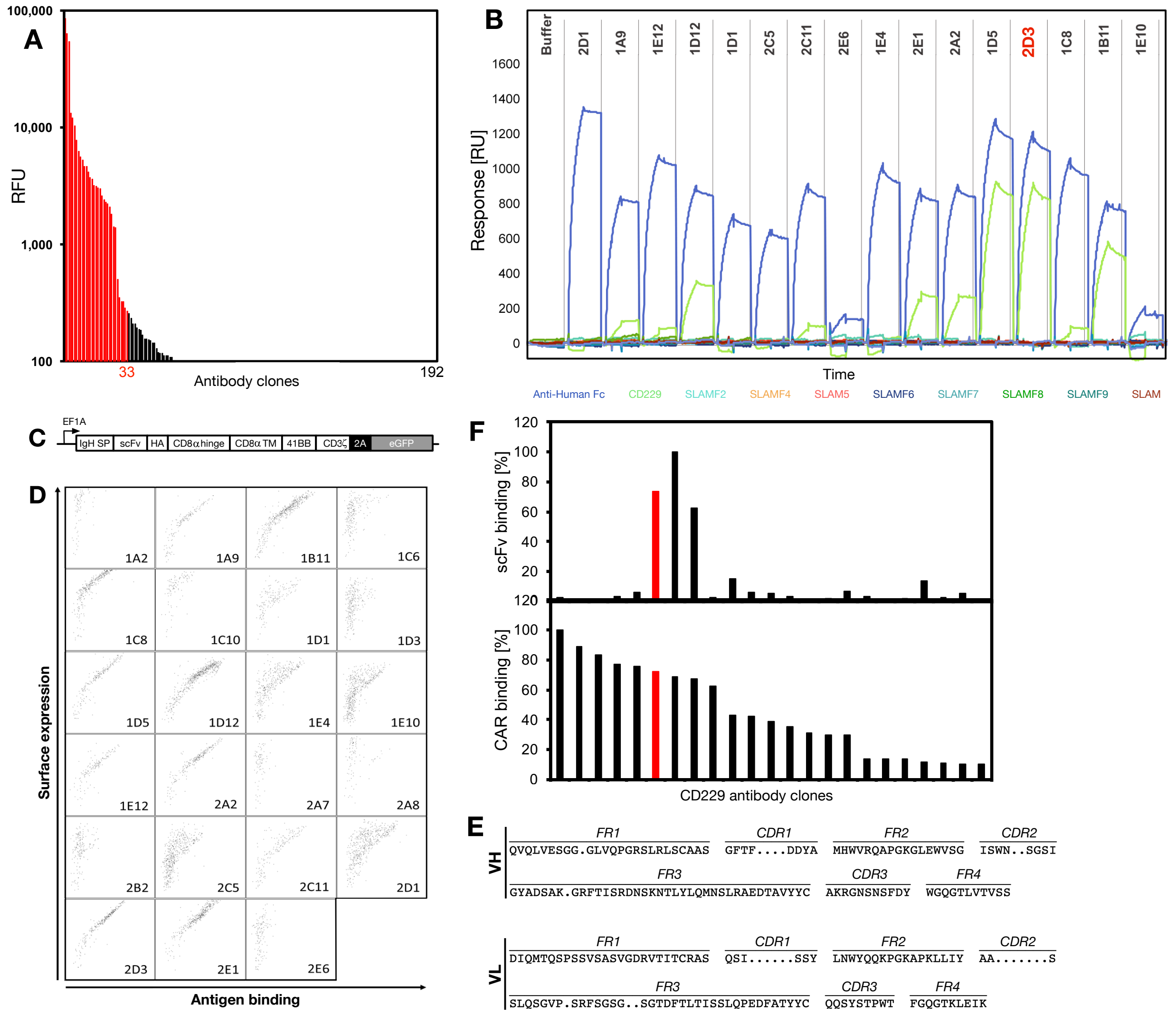

Supplementary Figure 1: Generation, screening, and validation of a fully-human CD229-specific monoclonal antibody. (A) Binding of 192 scFv antibody clones to recombinant human CD229 as determined by time-resolved fluorescence (TRF) assay. Red indicates clones selected for downstream sequencing. **(B)** Cross-reactivity of antibodies in scFv-Fc format to SLAM family receptors as determined by surface-plasmon resonance (SPR). **(C)** Schematic of CAR construct used in this study. **(D)** Surface expression and antigen binding of 23 CD229 CAR constructs as determined by staining of 293T cells transfected with single CAR constructs using an anti-HA antibody and fluorescently labelled recombinant human CD229 as determined by flow cytometry. **(E)** IMGT-gapped amino acid sequences of heavy (VH) an light (VL) variable domains of CD229-specific antibody clone 2D3. **(F)** Comparison of binding to recombinant CD229 by antibodies in scFv and CAR format as determined by TRF or flow cytometry expressed as the percentage of the best binder.

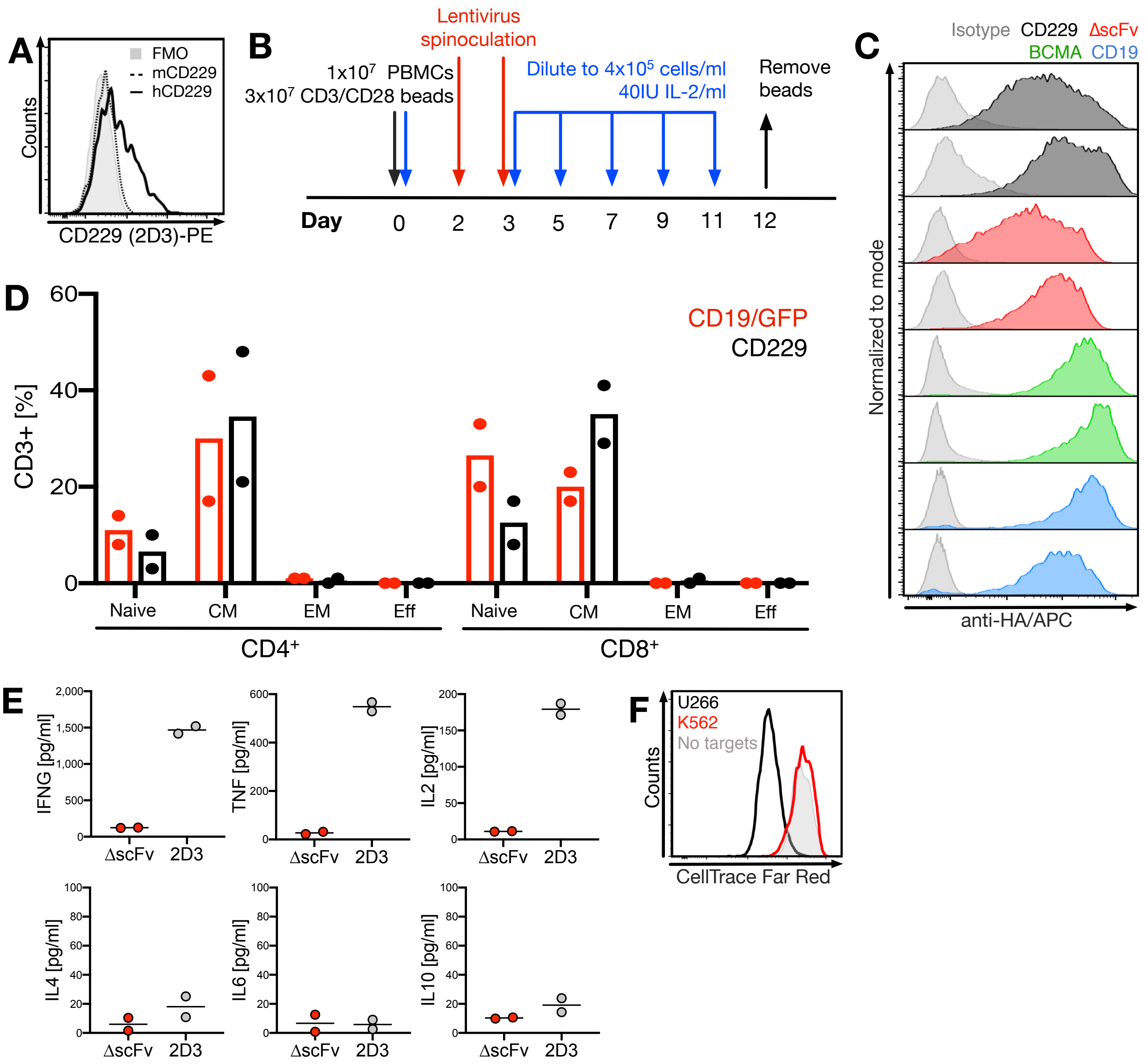

Supplementary Figure 2: Generation and characterization of CD229 CAR T cells. (A) Binding of 2D3 to 293T cells transduced with a human or mouse CD229 expression construct. Binding was determined using flow cytometry. **(B)** Schematic drawing showing the CD229 CAR T cell manufacturing process, adapted from Stroncek et. al. [1] **(C)** Surface expression of CAR constructs on primary T cells from two donors as determined by anti-HA staining using flow cytometry. **(D)** Phenotypes of CAR T cells from two healthy donors at the end of manufacturing as determined by flow cytometry. Naïve ($CD45RA^{+}CD45RO^{-}CD62L^{+}$), Eff = effector ($CD45RA^{+}CD45RO^{-}CD62L^{-}$), CM = central memory ($CD45RA^{-}CD45RO^{+}$ $CD62L^{+}$), EM = effector memory ($CD45RA^{-}CD45RO^{+}$ $CD62L^{-}$). Bars represent the mean from two donors. **(E)** T cell cytokine levels in the supernatant of co-cultures containing CD229-positive MM cell line U-266 and CD229 CAR T cells or T cells expressing a CAR without a binding domain (ΔscFv) as determined by cytometric bead array. Horizontal bars represent the mean of 2 technical replicates. **(F)** Proliferation of CD229 CAR T cells when co-cultured for 72 hours with CD229-positive U-266 cells, CD229-negative K562 cells, or without any target cells as determined by dilution of CellTrace Far Red dye.

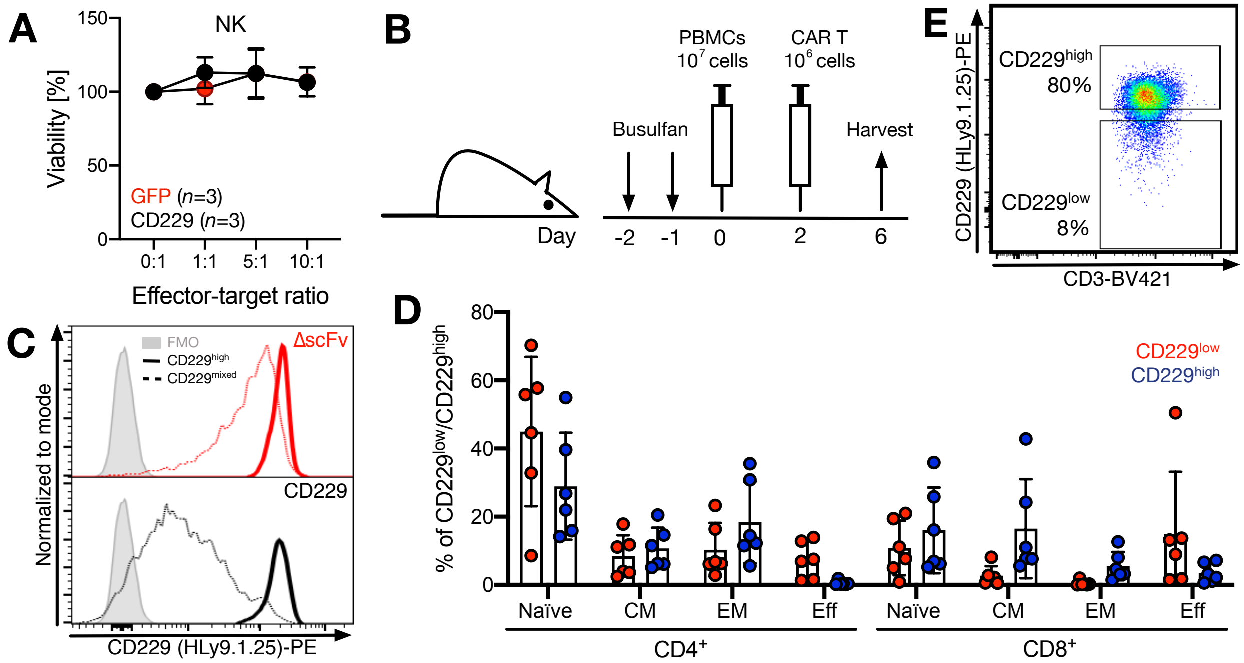

Supplementary Figure 3: T cell targeting by CD229 CAR T cells. (A) *In vitro* cytotoxicity of CD229 CAR T cells or GFP T cells against purified healthy NK cells stained with calcein AM as determined by a flow cytometry-based cytotoxicity assay. Data represent the mean ± standard deviation of 3 independent experiments. **(B)** Schematic drawing of the humanized mouse experiment setup. Three NSG mice per group were intraperitoneally injected with 20mg/kg busulfan for 2 consecutive days and afterwards intravenously injected with $1x10^7$ healthy peripheral blood mononuclear cells (PBMCs). Mice were sacrificed on day 6 after PBMC injection and peripheral blood and bone marrow analyzed for the presence of human PBMCs by flow cytometry. **(C)** T cells from a healthy donor were sorted for high CD229 expression ($CD229^{high}$) and labelled with CellTrace Far Red dye. Labelled $CD229^{high}$ cells were then combined with healthy unsorted T cells ($CD229^{mixed}$) and co-cultured over night with CD229 CAR T cells or ΔscFv CAR T cells at an effector-target ratio of 2:1. CD229 expression on labeled and unlabeled T cells from the same culture was determined by flow cytometry. **(D)** T cells from healthy donors were analyzed for differences in T cell phenotypes according to CD229 expression level using flow cytometry. Data represent the mean ± standard deviation from 6 healthy donors. Differences between $CD229^{neg/low}$ and $CD229^{high}$ T cell phenotypes were not statistically significant as determined by two-sided Student's t test correcting for multiple comparisons using the Sidak-Bonferroni method. Naïve ($CD45RA^+CD45RO^-CD62L^+$), Eff = effector ($CD45RA^+CD45RO^-CD62L^-$), CM = central memory ($CD45RA^-CD45RO^+$ $CD62L^+$), EM = effector memory ($CD45RA^-CD45RO^+$ $CD62L^-$). **(E)** Example of gating scheme for $CD229^{low}$ and $CD229^{high}$ T cells.

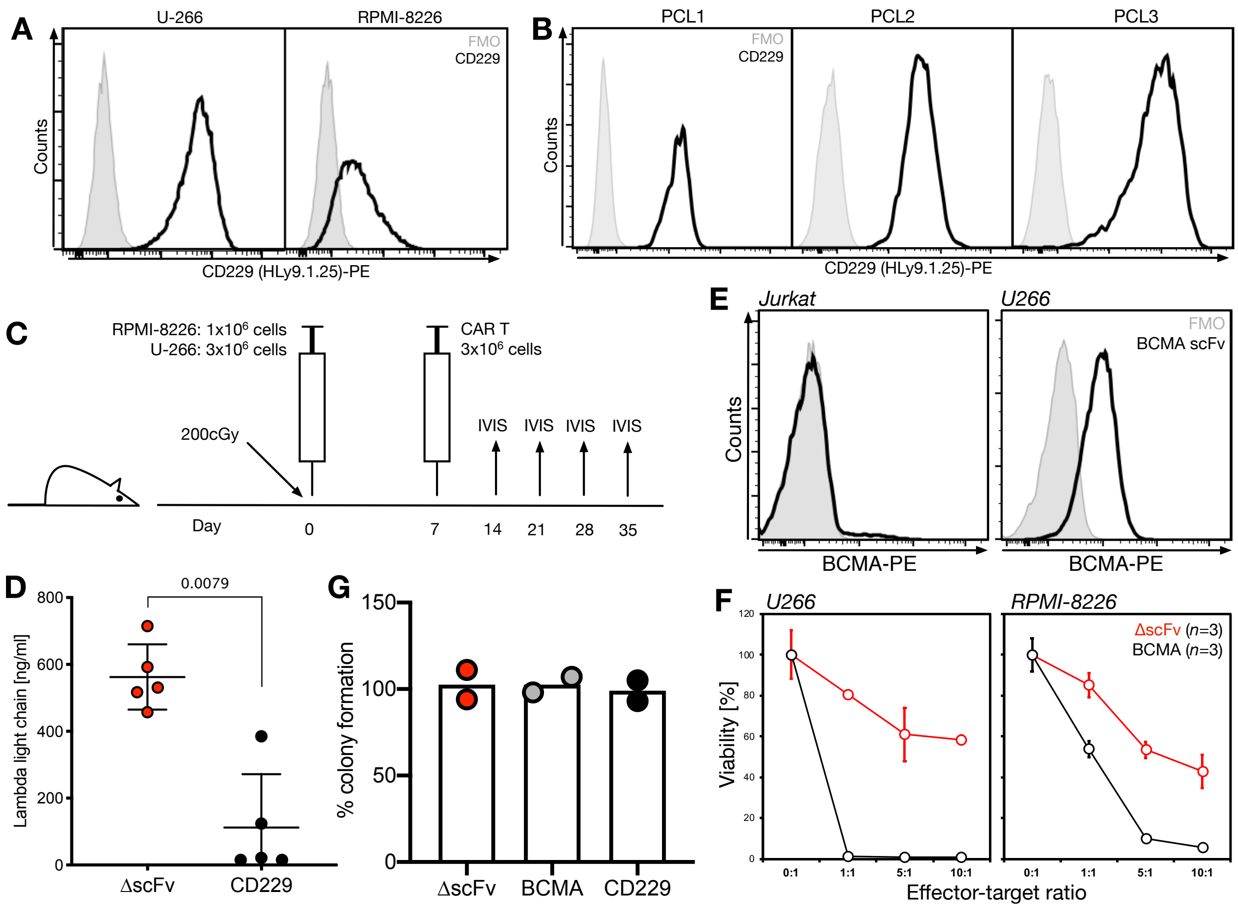

Supplementary Figure 4: Cytotoxic activity of CD229 and BCMA CAR T cells. (A) Expression of CD229 on the surface of multiple myeloma cell lines U-266 and RPMI-8226 as determined by flow cytometry. **(B)** Expression of CD229 on the surface of CD138+ tumor cells from 3 patients with plasma cell leukemia as determined by flow cytometry. **(C)** Schematic drawing of *in vivo* xenograft experiment. NSG mice were irradiated with 200cGy and injected with the indicated numbers of multiple myeloma cells expressing luciferase. After 1 week, mice were injected with CAR T cells and bioluminescence was determined weekly using IVIS. **(D)** Serum lambda light chain levels in animals that had received U-266 cells were determined by ELISA. Data represent the mean ± standard deviation from 5 animals per group. *p* value was determined by two-sided Student's t test. **(E)** Binding of the BCMA-specific scFv to BCMA+ U-266 cells but not to BCMA- Jurkat cells as determined by flow cytometry. **(F)** Cytotoxic activity of BCMA CAR T cells or ΔscFv CAR T cells against MM cell lines at different effector-target ratios as determined by a luciferase-based cytotoxicity assay. Data represent the mean ± standard deviation from 3 independent experiments. **(G)** Bone marrow mononuclear cells from 2 healthy donors were co-cultured with CD229, ΔscFv, or BCMA CAR T cells for 4-6 hours. Afterwards, samples were depleted of CD3+ CAR T cells and plated in methylcellulose. Colonies were counted after 14-21 days of culture. Bars represent the mean from 2 donors .

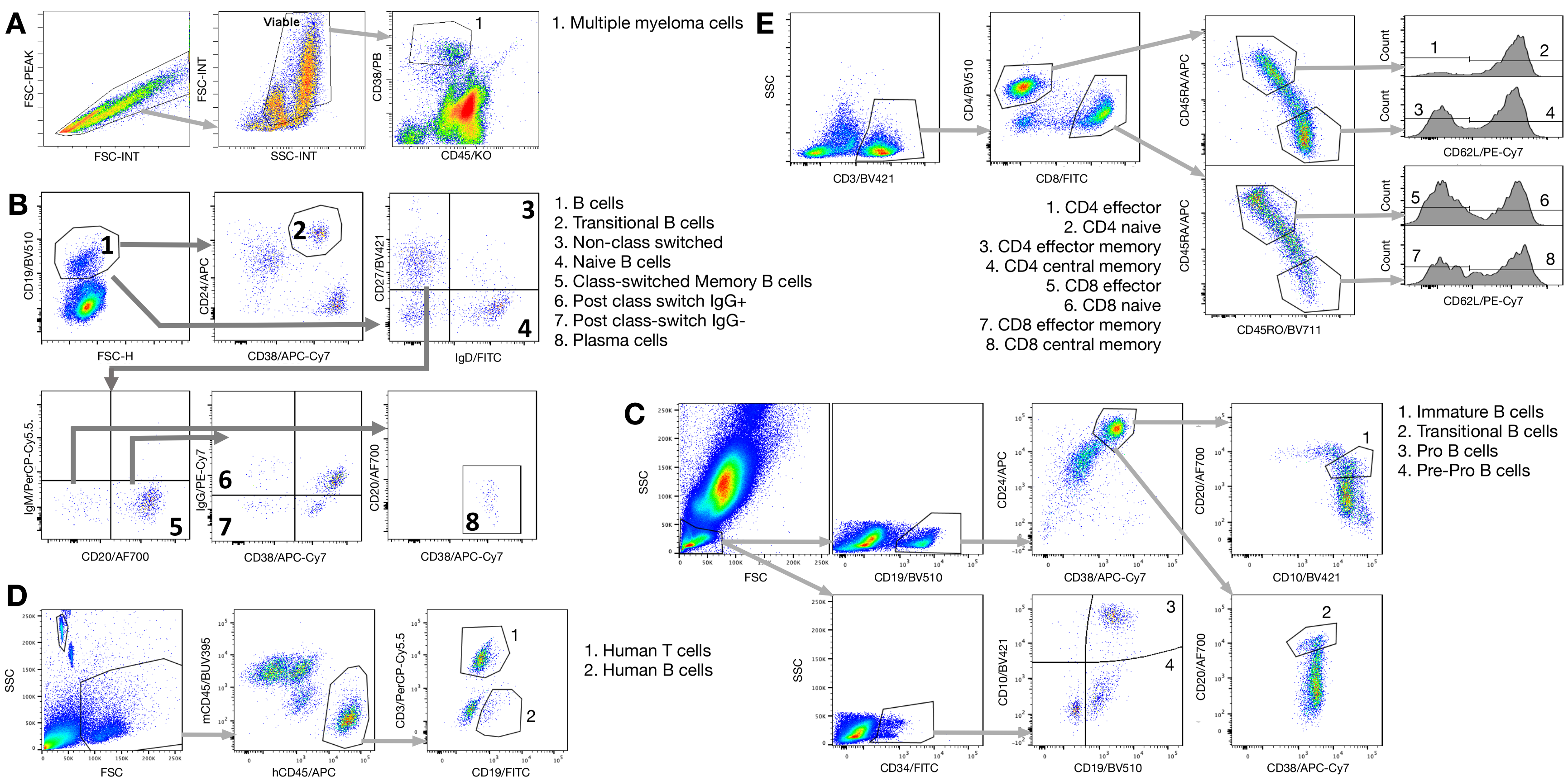

Supplementary Figure 5: Gating schema examples. (A) Example of gating schema to identify primary MM cells shown in Fig 1A and Fig. 2E. **(B)** Example of gating schema to identify B cell subsets shown in Fig. 1B and Fig. 4J. **(C)** Example of gating schema to identify Pro and Pre-Pro B cells shown in Fig. 1B. **(D)** Example of gating schema used to identify human T cells and B cells shown in Fig. 3H, Fig. 3J, Fig. 4G, and Fig. 4H. **(E)** Example of gating schema used to identify T cell subsets shown in Suppl. Fig. 2D.

SUPPLEMENTARY TABLES

Target	Clone	Supplier	Population
huCD229	HLy9.1.25	Biolegend/R&D Systems	Multiple
huCD3	UCHT1	Biolegend	Pan-T
huCD4	OKT5	Biolegend	T subset
huCD8	SK1	Biolegend	T subset
huCD10	HI10a	Biolegend	B lineage
huCD14	M5E2	Biolegend	Monocytes
huCD16	3G8	Biolegend	Neutrophils
huCD19	HIB19	Biolegend	MM/Pan-B
huCD20	2H7	Biolegend	B lineage
huCD24	ML5	Biolegend	B lineage
huCD27	M-T271	Biolegend	B subset
huCD34	8G12	BD	Hematopotic stem
huCD38	HB-7	Biolegend	MM/B
huCD45	2D1	Biolegend	Human leukocytes
mCD45	30-F11	BD	Mouse leukocytes
huCD45 RA	1834	Immunotech	T subset
huCD45 RO	UCHL1	Biolegend	T subset
huCD56	5.1H11	Biolegend	NK
huCD62L	DREG-56	Biolegend	T subset
huCD138	MI15	Biolegend	MM/Plasma
huCD197	G043H7	Biolegend	T subset
huCD269 (BCMA)	19F2	Biolegend	MM/B
huCD274	MIH4	BD	Tonic/activated T
Hemagglutinin	6E2	Cell Signaling Technology	CAR T
IgM	MHM-88	Biolegend	B lineage
IgD	IA6-2	Biolegend	B subset
HLA-DR	L243	Biolegend	Activated T
LIVE/DEAD Aqua	L34957	Life Technologies	Live/dead
Propidium iodide	421301	Biolegend	Live/dead
DAPI	D1306	Life Technologies	Live/dead
FLAG	M2	Sigma-Aldrich	CAR T

Supplementary Table 1: Table of monoclonal antibodies used for flow cytometry analyses.

REFERENCES

1. Stroncek, D.F., *et al.* Elutriated lymphocytes for manufacturing chimeric antigen receptor T cells. *J Transl Med* **15**, 59 (2017).
