# Supplementary material for: CD229 CAR T cells eliminate multiple myeloma and tumor propagating cells without fratricide
Source: Nat Commun. 2020 Feb 7;11:798. doi: 10.1038/s41467-020-14619-z (PMC7005855; doi:10.1038/s41467-020-14619-z)
Supplement: Supplementary file 3 — Description of Additional Supplementary Files [file 41467_2020_14619_MOESM3_ESM.pdf]

Description of Additional Supplementary Files

File Name: Supplementary Data 1
Description: Human membrane proteins analyzed for binding by 2D3 IgG as determined by flow cytometry.
